# Development of Incidence and Surgical Treatment of Penile Cancer in Germany from 2006 to 2016: Potential Implications for Future Management

**DOI:** 10.1245/s10434-021-10189-6

**Published:** 2021-06-12

**Authors:** Christer Groeben, Rainer Koch, Klaus Kraywinkel, Nina Buttmann-Schweiger, Martin Baunacke, Angelika Borkowetz, Christian Thomas, Johannes Huber

**Affiliations:** 1 Department of Urology, Medical Faculty Carl Gustav Carus, TU Dresden, Dresden, Germany; 2 National Center for Cancer Registry Data, Robert Koch Institute, Berlin, Germany

## Abstract

**Background:**

Penile cancer is a rare disease and surgical treatment often entails a significant impact on quality of life. The aim of this study was to analyze trends in surgical treatment patterns in Germany.

**Methods:**

We analyzed data from the nationwide German hospital billing database and the German cancer registry from 2006 to 2016. All penile cancer cases with penile surgery or lymph node dissection (LND) were included. We also analyzed the distribution of cases, extent of surgery, and length of hospital stay, stratified for annual caseload. The geographical distribution of centers for 2016 was presented.

**Results:**

During the investigated timespan, tumor incidences increased from 748 to 971 (*p* = 0.001). We identified 11,353 penile surgery cases, increasing from 886 to 1196 (*p* < 0.001), and 5173 cases of LND, increasing from 332 to 590 (*p* < 0.001). Cases of partial amputation increased from 45.8 to 53.8% (*p* < 0.001), while total amputation remained stable at 11.2%. Caseload in high-volume hospitals increased from 9.0 to 18.8% for penile surgery (*p* < 0.001) and from 0 to 13.1% for LND (*p* < 0.001). The increase in LND caseload was caused by an increase in inguinal LND, from 297 to 505 (*p* < 0.001), with increasing sentinel LND, from 14.2 to 21.9% (*p* = 0.098). The assessment of geographical distribution of cases in Germany revealed extensive areas without sufficient coverage by experienced centers.

**Conclusions:**

We saw consistent increases in penile surgery and LND, with a growing number of cases in high-volume hospitals, and, accordingly, an increase in tumor incidence. The increasing use of inguinal LND and organ-preserving surgery reflect the adaptation of current guidelines; however, geographical distribution of experienced centers could be improved.

**Supplementary Information:**

The online version contains supplementary material available at 10.1245/s10434-021-10189-6.

Penile cancer is a rare disease in Western countries, with a varying incidence from 0.4 to 1.8 per 100,000 cases (age-standardized rate [ASR]) and predominantly affecting men >60 years of age.^1^–^3^ About one-third of cases are attributed to past human papillomavirus (HPV) infections of the penile skin, whereas the remaining cases seem to be mostly generated by chronic inflammatory processes of the glans and prepuce.^4^ Since, after childhood circumcision, men show far lower incidences of penile cancer, removing the prepuce seems to have a preventive effect.^1^

When removed locally before metastasis or local progression, penile cancer can be safely cured, with only a little impact on the patients’ quality of life and sexual function. However, after local progression with vascular, corporal, or urethral invasion, mutilating surgery with partial or total amputation of the penis is the only reasonable option.^4^,^5^ In a locally advanced stage with > pT1b tumors or suspicion of lymph node metastasis, additional inguinal lymph node dissection (LND) should be performed according to the European Association of Urology guidelines for penile cancer,^6^ followed by pelvic LND if inguinal lymph nodes prove positive or in cases of radiographically suspicious pelvic lymph nodes. In cases of lymph node recurrence or extensive nodal metastasis, 5-year survival rates drop to ≤40%, depending on chemotherapy response.^7^ Despite currently ongoing studies with immune checkpoint inhibitors,^8^ data on possible effects are limited and, to date, prognosis for men with metastatic disease remains poor.^4^

Due to the low incidence of the disease, with about 900 newly diagnosed cases in Germany in 2014,^9^ experience with surgical and systemic treatment of penile cancer in about 330 urological clinics is expected to be rather low on average. However, numerous studies have proven a correlation between surgical management of malignant diseases and improved survival, as well as functional outcomes.^10^–^14^ For men with penile cancer requiring at least partial amputation of the penis, surgical experience is all the more important since skilled organ-preserving or reconstructive surgery can help to maintain quality of life and sexual function.^7^ Consequently, guidelines strongly recommend referring those patients to specialized centers.^6^,^15^ However, data on recent treatment patterns for the surgical management of penile cancer in Germany are lacking.

The aim of this study was to assess current trends of penile cancer surgery and LND in Germany. These developments should be analyzed with regard to the overall disease incidence and the regional distribution of care providers in order to derive possible optimizations.

## Methods

### Data Sources

The nationwide hospital billing database of the German Federal Statistical Office was used as the primary data source. The data extraction and cohort identification methods have been described in previous publications.^16^ The diagnosis is coded according to the standard International Classification of Diseases, Tenth Revision (ICD-10) coding system, while Operationen und Prozedurenschluessel (OPS), a German version of the International Classification of Procedures in Medicine, is implemented for procedures. The database is composed by the annual hospital billing data sets being transferred, according to legal obligation by German hospitals, to the Federal Statistical Office. The data are virtually complete for the given purpose.

Inclusion criteria were a diagnosis of penile cancer (ICD-10: C.60), uncertain neoplasm of the penile skin (D.407) or Bowen’s disease (D.074) combined with either penile surgery (OPS: 5-641, 5-642) or inguinal LND (5-401.5, 5-401.a, 5-402.4, 5-402.9, 5-404.h) or pelvic LND (OPS: 5-401.4, 5-401.9, 5-402.3, 5-402.5, 5-402.8, 5-404.f, 5-404.g). The surgical approach of penile surgery was grouped as excision or destruction of the primary tumor (OPS: 5-641), or partial (OPS: 5-642.0) or total amputation (OPS: 5-642.1, 5-642.2). Pelvic LND could be divided into an open (OPS: 5-401.4, 5-402.3, 5-402.5, 5-404.f) or laparoscopic approach (OPS: 5-401.9, 5-402.8, 5-404.g). The extent of inguinal LND was defined through ICD coding as sentinel (OPS: 5-401.5, 5-401.a), modified/reduced bilateral (OPS: 5-402.4, 5-402.9), or radical (OPS: 5-404.h). Additional assessment of surgical revision or complication management was performed using OPS codes 8-159.2, 8-148, and 8-149 (drainage of a lymphocele), OPS code 5-408 (lymphocele resection), or OPS codes 5-894, 5-895, 5-896, and 5-869.1 (treatment for wound-healing disorders).

The existing database was supplemented with additional institutional characteristics (i.e. teaching status, hospital size, and location). Annual hospital caseload categories were defined as low (< 4), medium (4–9), and high (≥ 10) according to our previous work in less frequent entities.^17^

We supplemented estimates on the nationwide incidence of penile cancer from the German National Centre for Cancer Registry Data at the Robert Koch Institute,^18^ which are presented in absolute numbers and as age-standardized incidence rates (old European standard population). We further used data from 14 (of 16) German cancer registries, representing 79% of the German male population whose data were available for the whole study period, to calculate tumor stage distribution and cases of histological types of penile cancer.

For identification of national providers and geographical localization, data from QB-Monitor 2016 and EasyMap (Lutum + Tappert DV-Beratung GmbH, Bonn, Germany) were implemented. Herein, we conducted a systematic search for cases with OPS codes of inguinal LND and penile (partial) amputation (see above). Non-urological cases were excluded from the analysis.

### Statistics

Rates, means, and trends were compared using the correlation coefficient and Wald tests. Rates and percentages of absolute values were predominantly used, and rates of relative values are distinctly specified. For trend analysis over time, F-tests of the linear regression coefficient of the annual caseload development were used. A *p*-value < 0.05 was regarded as significant. For statistical analysis SAS 9.4 (SAS Institute GmbH, Heidelberg, Germany) was used.

The included data were derived from fully anonymized databases with a high level of data protection. We followed the REporting of studies Conducted using Observational Routinely collected health Data statement (RECORD).^19^

## Results

### Epidemiology

**Fig. 1:**
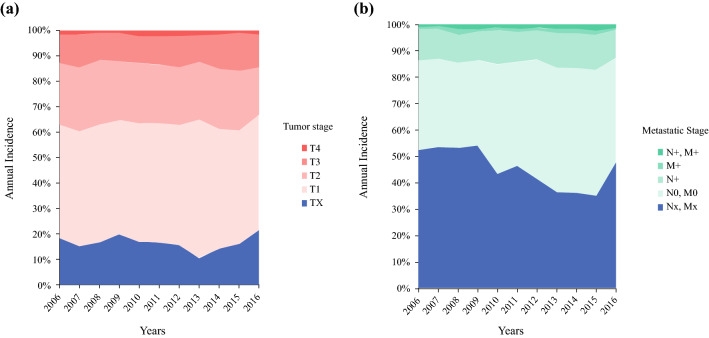
Development of tumor stages (**a**) and lymph node stages and other metastasis (**b**) at first diagnosis for penile cancer in Germany (2006–2016; 60% of the total population). *N+, M+* lymph node and other metastasis, *M+* other metastasis, lymph node-negative, *N+* lymph node metastasis only, *N0, M0* no metastasis, *Nx, Mx* metastasis unclear

According to the cancer registry data, the absolute incidence of penile cancer in Germany increased steadily from 748 cases in 2006 to 971 cases in 2016 (*p* < 0.001 for trend analysis); however, the ASR remained fairly stable between 1.4 and 1.6/100,000 cases during the investigated timespan (*p* = 0.15). The mean age of penile cancer patients increased from 67.2 ± 11.9 years in 2006 to 69.3 ± 12.1 years in 2016 (*p* = 0.001). Overall, 14.4% of patients presented with lymph node metastasis and 2.9% of patients presented with distant metastasis at primary diagnosis. Primary histology was squamous cell carcinoma in 92.6% of cases, melanoma in 1.1%, adenocarcinoma in 0.9%, basal cell carcinoma in 0.8%, and not specified penile malignancies in 4.6% of cases. Figure 1 shows the development of the incidence of tumor stages and metastatic disease.

### Penile Cancer Surgery

**Fig. 2:**
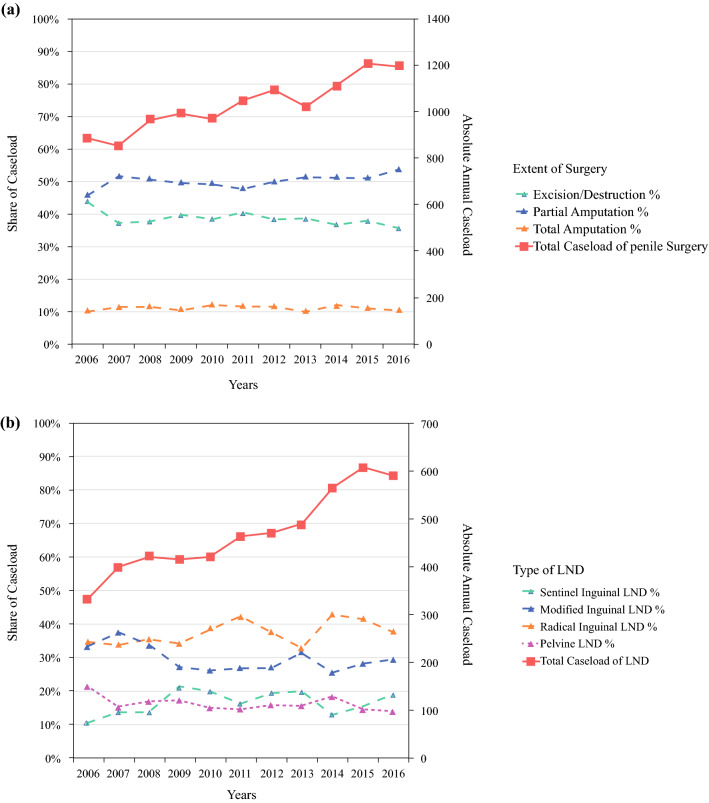
Extent and absolute number of penile surgeries (**a**) and LNDs for penile cancer (**b**) in Germany (2006–2016). *LND* lymph node dissection

A total of 11,353 cases of penile surgery for penile carcinoma were extracted from the DRG database, with increasing annual numbers from 886 in 2006 to 1196 in 2016 [*p* < 0.001]. The average number of hospitals was 330.2 per year, increasing from 311 in 2006 to 350 in 2016. Patient characteristics and patterns of care for penile surgery and LND are presented in Table 1. Mean patient age increased from 66.1 ± 13.0 years in 2006 to 68.5 ± 12.5 years in 2016 (*p* < 0.001). Figure 2 demonstrates the trends in caseload and surgical extent. Cases of partial amputation increased from 45.8% to 53.8% (*p* < 0.001), while total amputation remained stable at 11.2% and local excision/destruction declined from 43.9 to 35.7% (*p* = 0.035). A laser was used in 7.1% of cases. Primary lesions were situated at the prepuce in 7.2% of cases, glans in 44.8%, and shaft in 5.6% of cases, with overlapping or uncertain location in 6.1% and 7.0% of cases, respectively.

**Table 1:** Patient characteristics and patterns of care of penile cancer surgery and LND in Germany (2006–2016)

	Penile surgery	LND
Total number of cases	11,353	5173
Age, years [mean ± SD]	67.3 ± 12.5	64.5 ± 11.4
*Annual hospital caseload*		
< 4	4446 (39.2)	2818 (54.5)
4–9	5428 (47.8)	1991 (38.5)
> 9	1479 (13.0)	364 (7.0)
*Teaching status*		
Academic	3110 (27.4)	3525 (68.1)
*Size of hospital, no. of beds*		
< 300	2194 (19.3)	870 (16.8)
301–800	4820 (42.5)	2037 (39.4)
> 800	4339 (38.2)	2266 (43.8)
*Surrounding city size, no. of inhabitants*		
< 20,000	1708 (15.0)	714 (13.8)
20,001–100,000	3835 (33.8)	1721 (33.3)
100,001–500,000	3373 (29.7)	1632 (31.5)
> 500,000	2437 (21.5)	1106 (21.4)

Data are expressed as *n* (%) unless otherwise specified

*LND* lymph node dissection, *SD* standard deviation

**Fig. 3:**
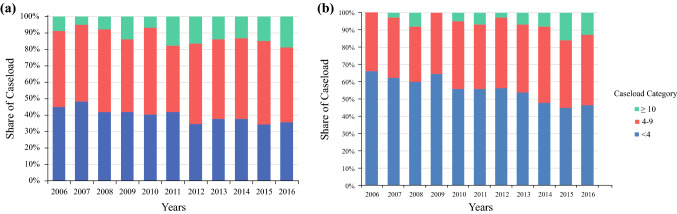
Distribution of patients with penile surgery (**a**) and LND (**b**) in Germany, stratified for annual hospital caseload categories (2006–2016). *LND* lymph node dissection

Figure 3a shows the volume of penile cancer surgery according to the hospital caseload category. The share of patients treated in hospitals with high volume increased from 9.0% in 2006 to 18.8% in 2016, but decreased in hospitals with an annual caseload of < 4, from 44.5 to 35.3% (*p* = 0.001 for trend comparison).

### Lymph Node Dissection

Overall, 5173 cases of LND for penile carcinoma were included for analysis, increasing considerably from 332 in 2006 to 590 in 2016 (*p* < 0.001). The mean age at LND increased from 63.0 ± 11.6 years to 65.4 ± 11.3 years (*p* < 0.001). The increase in caseload was mainly caused by an increase in inguinal LNDs, from 297 to 505 (*p* < 0.001). The share of pelvic LNDs remained stable at 16.1% of cases (average of 17.5% laparoscopically). There was a non-significant increase, from 14.2% in 2006 to 21.9% in 2016 (*p* = 0.098), for applying the sentinel technique with inguinal LND. Figure 3b presents the caseload distribution for LND according to the hospital caseload category. The share of patients treated in high-volume hospitals increased from 0% in 2006 to 13.1% in 2016 (*p* < 0.001), but decreased in low-volume hospitals, from 66.0 to 46.3% (*p* = 0.008). A mean of 95.8% of cases were performed in urological departments. Overall, 64.9% of hospitals performing penile cancer surgery did not perform inguinal LND for penile cancer, increasing from 49.8 to 72.3% (*p* < 0.001). The rate of surgical revision was 11.1%, increasing from 6.3 to 12.5% (*p* < 0.001), independent of hospital caseload. Predominant were wound-healing disorders at 49.6%, followed by percutaneous lymphocele drainage (34.6%) and lymphocele resection (15.8%). The overall length of stay (LOS) for LND was 14.2 ± 11.5 days, decreasing from 15.4 ± 10.5 in 2006 to 13.2 ± 12.6 days in 2016 (*p* = 0.005). LOS was shorter in high-volume hospitals versus low-volume hospitals (13.0 ± 11.8 days vs. 15.0 ± 12.2 days) [*p* = 0.002]. Patients with sentinel LND had a shorter LOS (12.4 ± 12.5 days) than patients with modified LND (14.2 ± 11.21 days) and radical LND (15.6 ± 12.9 days) [*p* < 0.001].

**Fig. 4:**
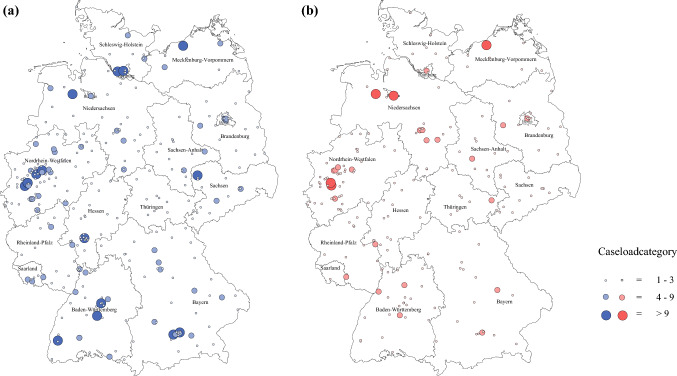
Distribution of (partial) penile amputation (**a**) and inguinal LND (**b**) in urologic departments for the year 2016. *LND* lymph node dissection

Figure 4 demonstrates the distribution of cases of inguinal LND and penile amputation (partial or radical) in Germany, on a geographic map, for the year 2016.

## Discussion

From 2006 to 2016, the annual incidence of penile cancer increased steadily (29.8%), in accordance with the caseload for penile cancer surgery (35.0%) and LND (53.3%). Likewise, the share of cases being performed in hospitals with high caseloads increased for both penile surgery and LND. The increase in LND caseload was mainly caused by increasing inguinal LND numbers. Inguinal LND was predominantly performed in a radical or modified fashion, with a slight trend towards an increasing use of the sentinel technique.

### Trends of Newly Diagnosed Penile Cancer in Germany

In absolute numbers, the incidence of penile cancer in Germany has increased steadily, by nearly one-third, during the investigated timespan; however, the age-adjusted incidence rate remained stable. Therefore, the increasing incidence is mainly explained by the demographic shift, with an increasing share of older male citizens in the German population.^20^ At the same time, there was no change in tumor stage distribution and the share of metastatic disease (Fig. 2). When compared with penile cancer epidemiology in the current literature, we see similar basic incidence rates in other European populations;^21^ however, the incidence rate varies in different countries and is higher in some northern European countries, e.g. Sweden.^4^,^22^ Compared with contemporary results from the US, the German incidence rate is higher, although current studies demonstrated significant differences in the US itself depending on patient ethnicity, differences in religious practice (i.e. childhood circumcision), and socioeconomic status.^23^ Histologic distribution in the US is also comparable with Germany, with approximately 93% of squamous cell carcinomas, and small percentages of melanoma, adenocarcinoma, and basal cell carcinoma.^4^,^23^

### Trends of Penile Surgery and Lymph Node Dissection for Penile Cancer in Germany

The total number of penile surgeries for penile cancer increased by approximately one-third during the 11-year timespan of this study, which can be primarily explained by the increasing incidence of penile cancer. Nevertheless, our analysis also demonstrated that partial amputation is used in an increasing percentage of cases, following the guideline-recommended principle of organ preservation.^6^,^15^ Nonetheless, this might lead to repeat surgery instead of one-time radical treatment.^4^ Additionally, the number of hospitals performing surgery for penile carcinoma increased by 12.5%. Moreover, the overall annual caseload per hospital increased, leading to a higher number of cases being performed in hospitals with a high caseload (Fig. 3) and thus to presumably improved expertise in the respective centers. This trend is even surpassed by the increases in total caseload of LND for penile cancer (>50%), and, interestingly, was almost entirely caused by an increase in inguinal LNDs, of which we noticed a nonsignificant increase in the percentage of sentinel inguinal LNDs (from 14 to 22%), as recommended by the European Association of Urology guidelines.^6^

A positive correlation between high caseload volume and better postoperative outcomes has been repeatedly shown in several major procedures such as radical prostatectomy and radical cystectomy.^10^,^12^–^14^,^24^ Technically challenging surgical procedures such as penile reconstruction after partial amputation, as well as inguinal LND, also require high levels of experience;^4^ however, important endpoints such as cosmetics, functional outcomes, and quality of life were not available in the presented datasets. LOS was about 2 days shorter in high-volume hospitals, although this endpoint was not a sufficient surrogate for relevant outcomes. Due to the low overall incidence rates and the increasing number of hospitals performing penile surgery, the majority of cases are still performed in hospitals in which fewer than 10 procedures are performed annually; in 2016, this resulted in a rate of 81% for penile surgery and 87% for LND. National health policy making could enforce the centralization of oncological care for penile cancer in general and for surgical treatment in particular. One option could be the implementation of minimum caseload requirements, as already applied in Germany since 2004, for selected surgical procedures and treatments, with limited effect to date.^25^ Another option could be the certification of specialized penile cancer centers, with several European countries having established specialized centers for penile cancer, e.g. the Scandinavian countries.^4^,^26^ or the UK^21^,^27^ The advantages of centralized treatment were shown for the timely referral from diagnosis to treatment,^26^ accuracy of the pathological assessment,^21^ and survival rates.^27^ Furthermore, improved guideline adherence associated with more frequent lymph node staging was shown in European countries as well as in the US.^22^,^27^,^28^ Therefore, an accelerated diagnostic process, and treatment according to guidelines, could be selling points for the centralization of care for penile cancer.

### Geographical Distribution and Centralization Tendencies

Since penile cancer has a high incidence in older men with potentially reduced mobility, being able to reach a medical center with adequate experience with reasonable effort is of high importance. Therefore, we demonstrated the geographical distribution of centers, along with their surgical caseload, for the year 2016 (Fig. 4). The results showed a vast distribution of penile (partial) amputation as well as inguinal LND throughout Germany, with concentration to several centers in the north and west (5 for LND, 15 for penile surgery). On the one hand, this demonstrates the adequate implementation of guideline-requested invasive lymph node staging,^6^,^15^ while on the other hand, especially in the rural areas of federal states with a larger geographical extension (e.g. Bavaria, Brandenburg, Lower Saxony), extensive areas exist without hospitals with adequate experience in penile cancer surgery. For respective patients, the next experienced urologic center can be located several hours away. Health policy measures with defining regional centers could concentrate the available caseload to institutions with an equal geographical distribution, and thereby create further experienced providers of penile cancer care.

### Limitations and Strengths

Our study was the first to analyze treatment patterns of penile surgery and LND for penile cancer in Germany using total population data covering 11 years to depict possible developments over time. Adding national cancer registry data and the regional distribution of penile cancer care providers complemented these total population data to draw a more complete picture of the German situation. Our study focused exclusively on surgical treatment, however systemic treatment and radiotherapy are also important cornerstones in the treatment of penile carcinoma, but were not included in our analysis. The main study limitations lie in the nature of the data itself. Although billing data are highly accurate, detailed information on tumor and patient characteristics is not available. Due to data protection regulations, single patients or institutions may not be identified from the German DRG database. Therefore, revision and verification of each data set is not possible. Due to the data structure, additional hospital stays or outpatient treatment of the same patient are not assignable and outcomes can only be determined for the duration of the inpatient stay. Therefore, patient-reported outcome measures and survival outcomes are missing. This information is essential to provide the rationale for designating certain institutions as ‘quality centers’. Finally, for the analyses of tumor stage distribution and histology, data from 2 of 16 German federal states were excluded as these registries did not cover the whole study period. Therefore, the presented results rely on 79% of the estimated incidence of penile cancer cases. Given the extensive caseload numbers for this rare procedure, some slight variances and small irregularities appear to be negligible; however, the principal risk of systematic bias has to be kept in mind when interpreting the results.

## Conclusions

We saw consistent increases in caseload numbers for penile surgery and LND, in relation to penile cancer in Germany, of 35% and 53%, respectively, over an 11-year period, and, accordingly, an increase in penile cancer incidence by approximately 30%. Surgical experience increased in the respective hospitals, along with the rising caseload numbers. Increasing numbers of inguinal LNDs and organ-preserving surgeries reflect the ongoing adaptation of current guideline recommendations. Nevertheless, geographical distribution of experienced centers in Germany could be improved by respective health policy-making in order to provide patients with adequate treatment in their regional areas.

## Supplementary Information

Below is the link to the electronic supplementary material.

**Supplementary Table 1** Hospitals and caseload of penile amputation for the year 2016.

**Supplementary Table 2** Hospitals and caseload of inguinal LND for penile cancer for the year 2016.

## Data Availability

German Research Data Center of the Federal Statistical Office, DRG statistics from 2006 to 2016, German National Centre for Cancer Registry Data (Robert Koch Institute), and our own calculations.
