# Supplementary material for: Development of Incidence and Surgical Treatment of Penile Cancer in Germany from 2006 to 2016: Potential Implications for Future Management
Source: Ann Surg Oncol. 2021 Jun 12;28(13):9190–8. doi: 10.1245/s10434-021-10189-6 (PMC8591000; doi:10.1245/s10434-021-10189-6)
Supplement: Supplementary file 1 — Supplementary Table 1 Hospitals and caseload of penile amputation for the year 2016. [file 10434_2021_10189_MOESM1_ESM.docx]

**Supplementary Table 2:** Hospitals and caseload of inguinal LND for penile cancer for the year 2016.

| Name of hospital | City | Total caseload of inguinal LND |
| --- | --- | --- |
| Universitätsmedizin Rostock - Teilkörperschaft der Universität Rostock | Rostock | 29 |
| Klinikum Oldenburg AöR | Oldenburg | 11 |
| Universitätsklinikum Düsseldorf | Düsseldorf | 11 |
| Klinikum Bremen-Mitte | Bremen | 10 |
| Paracelsus Klinik Düsseldorf Golzheim | Düsseldorf | 10 |
| Charité - Universitätsmedizin Berlin | Berlin | 9 |
| Universitätsklinikum Tübingen | Tübingen | 8 |
| SLK-Kliniken Heilbronn GmbH - Klinikum am Gesundbrunnen | Heilbronn | 7 |
| HELIOS Klinikum Salzgitter GmbH | Salzgitter | 6 |
| KRH Klinikum Siloah | Hannover | 6 |
| Universitätsmedizin der Johannes Gutenberg-Universität Mainz | Mainz | 6 |
| Städtisches Klinikum Karlsruhe | Karlsruhe | 6 |
| Klinikum rechts der Isar der Technischen Universität München | München | 6 |
| SRH Wald-Klinikum Gera GmbH | Gera | 6 |
| Universitätsklinikum Hamburg-Eppendorf | Hamburg | 5 |
| St. Bernward Krankenhaus | Hildesheim | 5 |
| Evangelisches Klinikum Niederrhein gGmbH | Oberhausen | 5 |
| Universitätsklinikum Köln | Köln | 5 |
| St. Barbara-Hospital | Gladbeck | 5 |
| Knappschaftskrankenhaus Bottrop GmbH | Bottrop | 5 |
| Klinikum Dortmund Nord | Dortmund | 5 |
| Caritas-Krankenhaus St. Josef | Regensburg | 5 |
| Universitätsklinikum des Saarlandes | Homburg | 5 |
| Städtisches Klinikum Brandenburg GmbH | Brandenburg an der Havel | 5 |
| Vinzenzkrankenhaus Hannover | Hannover | 4 |
| AMEOS Klinikum Aschersleben-Staßfurt GmbH | Aschersleben | 4 |
| Prosper-Hospital gGmbH | Recklinghausen | 3 |
| Klinikum Lippe Detmold | Detmold | 3 |
| Ortenau Klinikum Offenburg-Gengenbach Standort Ebertplatz | Offenburg | 3 |
| Klinikum der Universität München | München | 3 |
| HELIOS Klinikum Pirna | Pirna | 3 |
| UNIVERSITÄTSKLINIKUM Schleswig-Holstein, Campus Kiel | Kiel | 2 |
| Kath. Marienkrankenhaus gemeinnützige GmbH | Hamburg | 2 |
| Städtisches Klinikum Braunschweig gGmbH | Braunschweig | 2 |
| Klinikum Wolfsburg | Wolfsburg | 2 |
| Helios Klinik Cuxhaven GmbH | Cuxhaven | 2 |
| Ammerland-Klinik Westerstede | Westerstede | 2 |
| Kliniken Essen-Mitte Evang. Huyssens-Stiftung 01 | Essen | 2 |
| Krankenhaus St. Franziskus | Mönchengladbach | 2 |
| [2017-09-27 17:15:22] Städtisches Klinikum Solingen gemeinnützige GmbH | Solingen | 2 |
| Uniklinik RWTH Aachen | Aachen | 2 |
| Betriebsteil Bardenberg | Würselen-Bardenberg | 2 |
| Herz-Jesu-Krankenhaus Hiltrup GmbH | Münster | 2 |
| St. Elisabeth-Krankenhaus | Ibbenbüren | 2 |
| Augusta-Kranken-Anstalt gGmbH Bochum | Bochum | 2 |
| Marien Hospital Herne, Klinikum der Ruhr-Universität Bochum | Herne | 2 |
| Knappschaftskrankenhaus Dortmund, Klinikum Westfalen GmbH | Dortmund | 2 |
| Diakonie Klinikum Ev. Jung-Stilling-Krankenhaus | Siegen | 2 |
| Asklepios Paulinen Klinik | Wiesbaden | 2 |
| AGAPLESION MARKUS KRANKENHAUS | Frankfurt am Main | 2 |
| Asklepios Stadtklinik Bad Wildungen | Bad Wildungen | 2 |
| Gemeinschaftsklinikum Mittelrhein, Kemperhof | Koblenz | 2 |
| Diakonie-Klinikum Stuttgart | Stuttgart | 2 |
| Klinikum Ludwigsburg | Ludwigsburg | 2 |
| Diakonie-Klinikum Schwäbisch Hall gGmbH | Schwäbisch Hall | 2 |
| Klinikum Stuttgart - Katharinenhospital (KH) und Olgahospital / Frauenklinik (OH) | Stuttgart | 2 |
| Universitätsklinikum Mannheim GmbH | Mannheim | 2 |
| Klinikum am Steinenberg | Reutlingen | 2 |
| Waldkrankenhaus St. Marien | Erlangen | 2 |
| Klinikum Fürth | Fürth | 2 |
| Vivantes Humboldt-Klinikum | Berlin | 2 |
| Ruppiner Kliniken | Neuruppin | 2 |
| Klinikum Ernst von Bergmann gemeinnützige GmbH | Potsdam | 2 |
| Evangelisches Krankenhaus Luckau gGmbH | Luckau | 2 |
| Klinikum Frankfurt (Oder) GmbH | Frankfurt (Oder) | 2 |
| Universitätsmedizin Greifswald - Körperschaft des öffentlichen Rechts | Greifswald | 2 |
| HELIOS Kliniken Schwerin | Schwerin | 2 |
| Zeisigwaldkliniken Bethanien Chemnitz | Chemnitz | 2 |
| Heinrich-Braun-Klinikum gemeinnützige GmbH, Standort Zwickau | Zwickau | 2 |
| Klinikum St. Georg gGmbH | Leipzig | 2 |
| Städtisches Klinikum Dresden - Standort Friedrichstadt | Dresden | 2 |
| HELIOS Klinikum Aue | Aue | 2 |
| KLINIKUM MAGDEBURG gemeinnützige GmbH | Magdeburg | 2 |
| Universitätsklinikum Halle (Saale) | Halle (Saale) | 2 |
| AMEOS Klinikum Halberstadt | Halberstadt | 2 |
| HELIOS Klinik Blankenhain | Blankenhain | 2 |
| HELIOS Klinikum Erfurt | Erfurt | 2 |
| Diakonissenkrankenhaus Flensburg | Flensburg | 1 |
| Sankt Elisabeth Krankenhaus Kiel | Kiel | 1 |
| Regio Kliniken GmbH - Klinikum Wedel | Wedel | 1 |
| AK SEGEBERGER KLINIKEN GMBH | Bad Segeberg | 1 |
| Klinikum Itzehoe | Itzehoe | 1 |
| FEK - Friedrich-Ebert-Krankenhaus Neumünster GmbH | Neumünster | 1 |
| Sana Kliniken Lübeck GmbH | Lübeck | 1 |
| Josef-Hospital Delmenhorst Krankenhaus gGmbH | Delmenhorst | 1 |
| Universitätsmedizin Göttingen | Göttingen | 1 |
| Evangelisches Krankenhaus Göttingen-Weende gGmbH | Göttingen | 1 |
| DIAKOVERE Friederikenstift | Hannover | 1 |
| Medizinische Hochschule Hannover | Hannover | 1 |
| KRH Klinikum Robert Koch Gehrden | Gehrden | 1 |
| KRH Klinikum Großburgwedel | Burgwedel | 1 |
| AMEOS Klinikum Seepark Geestland | Geestland | 1 |
| Städtisches Klinikum Lüneburg gemeinnützige GmbH | Lüneburg | 1 |
| Klinikum Osnabrück GmbH | Osnabrück | 1 |
| Universitätsklinikum Essen | Essen | 1 |
| Alfried Krupp Krankenhaus Steele | Essen | 1 |
| HELIOS Klinikum Krefeld | Krefeld | 1 |
| Malteser Krankenhaus St. Josefshospital Uerdingen | Krefeld | 1 |
| [2017-10-02 11:07:06] Alexianer Krefeld GmbH - Krankenhaus Maria-Hilf | Krefeld | 1 |
| Städtische Kliniken Mönchengladbach GmbH | Mönchengladbach | 1 |
| HELIOS Klinikum Wuppertal GmbH | Wuppertal | 1 |
| Hospital zum Heiligen Geist Kempen GmbH & Co. KG | Kempen | 1 |
| Allgemeines Krankenhaus Viersen GmbH | Viersen | 1 |
| Marien-Hospital Wesel gGmbH | Wesel | 1 |
| Städtische Kliniken Neuss - Lukaskrankenhaus - GmbH | Neuss | 1 |
| Universitätsklinikum Bonn | Bonn | 1 |
| Malteser Krankenhaus Seliger Gerhard Bonn/Rhein-Sieg | Bonn | 1 |
| Johanniter GmbH - Waldkrankenhaus Bonn | Bonn | 1 |
| Kliniken der Stadt Köln gGmbH - Krankenhaus Holweide | Köln | 1 |
| St. Elisabeth-Krankenhaus Köln GmbH | Köln | 1 |
| St.-Antonius-Hospital | Eschweiler | 1 |
| Krankenhaus Düren gem. GmbH | Düren | 1 |
| St.-Katharinen-Hospital GmbH | Frechen | 1 |
| Marien-Krankenhaus Bergisch Gladbach | Bergisch Gladbach | 1 |
| GFO Kliniken Troisdorf, Betriebsstätte St. Josef Troisdorf | Troisdorf | 1 |
| Universitätsklinikum Münster | Münster | 1 |
| Ludgerus-Kliniken Münster GmbH / Raphaelsklinik | Münster | 1 |
| St. Antonius-Hospital Gronau GmbH | Gronau | 1 |
| Mathias-Spital Rheine / Gesundheitszentrum Rheine | Rheine | 1 |
| Franziskus Hospital Bielefeld | Bielefeld | 1 |
| St. Josefs-Hospital | Dortmund | 1 |
| St. Josef-Krankenhaus, Hamm Bockum-Hövel | Hamm | 1 |
| Klinikum Darmstadt GmbH | Darmstadt | 1 |
| Universitätsklinikum Gießen und Marburg, Standort Gießen | Gießen | 1 |
| Klinikum Wetzlar-Braunfels | Wetzlar | 1 |
| Sana Klinikum Offenbach GmbH | Offenbach | 1 |
| HELIOS Dr. Horst-Schmidt-Kliniken Wiesbaden | Wiesbaden | 1 |
| Klinikum Kassel GmbH | Kassel | 1 |
| Klinikum Fulda gAG | Fulda | 1 |
| Universitätsklinikum Gießen und Marburg GmbH, Standort Marburg | Marburg | 1 |
| DRK Krankenhaus Biedenkopf | Biedenkopf | 1 |
| Stadtkrankenhaus Korbach gGmbH | Korbach | 1 |
| GPR Klinikum | Rüsselsheim | 1 |
| Klinikum Idar-Oberstein GmbH | Idar-Oberstein | 1 |
| St. Nikolaus-Stiftshospital GmbH Andernach | Andernach | 1 |
| Evangelisches Krankenhaus, Betriebsstätte Dierdorf | Dierdorf | 1 |
| BundeswehrZentralkrankenhaus Koblenz | Koblenz | 1 |
| Krankenhaus der Barmherzigen Brüder Trier | Trier | 1 |
| Klinikum Worms gGmbH | Worms | 1 |
| Heilig-Geist-Hospital Bingen gGmbH | Bingen | 1 |
| medius KLINIK OSTFILDERN-RUIT | Ostfildern | 1 |
| Rems-Murr-Klinikum Winnenden | Winnenden | 1 |
| Kliniken Landkreis Heidenheim gGmbH | Heidenheim | 1 |
| Kliniken Sindelfingen | Sindelfingen | 1 |
| Caritas-Krankenhaus Bad Mergentheim | Bad Mergentheim | 1 |
| Universitätsklinikum Heidelberg | Heidelberg | 1 |
| Diakonissenkrankenhaus Mannheim | Mannheim | 1 |
| GRN Gesundheitszentren Rhein-Neckar gGmbH Kreiskrankenhaus Eberbach | Eberbach | 1 |
| Siloah St. Trudpert Klinikum | Pforzheim | 1 |
| Kliniken Nagold | Nagold | 1 |
| Loretto-Krankenhaus (RkK) Freiburg | Freiburg | 1 |
| Schwarzwald-Baar Klinikum Villingen-Schwenningen GmbH | Villingen-Schwenningen | 1 |
| Hegau-Bodensee-Klinikum Singen | Singen | 1 |
| Universitätsklinikum Freiburg | Freiburg | 1 |
| Universitätsklinikum Ulm | Ulm | 1 |
| Kreisklinik Ebersberg | Ebersberg | 1 |
| Urologische Klinik München - Planegg | Planegg | 1 |
| Klinikum Traunstein | Traunstein | 1 |
| Klinikum Ingolstadt GmbH | Ingolstadt | 1 |
| Städtisches Klinikum München GmbH, Klinikum Bogenhausen | München | 1 |
| Klinikum St. Elisabeth Straubing GmbH | Straubing | 1 |
| Klinikum Neumarkt | Neumarkt i.d.OPf. | 1 |
| Klinik Hohe Warte | Bayreuth | 1 |
| Klinikum Coburg GmbH | Coburg | 1 |
| Klinikum Nürnberg Nord | Nürnberg | 1 |
| Universitätsklinikum Würzburg | Würzburg | 1 |
| Missionsärztliche Klinik | Würzburg | 1 |
| Klinikum Augsburg mit Kliniken für Kinder und Jugendliche | Augsburg | 1 |
| Memmingen | Memmingen | 1 |
| SHG-Kliniken Völklingen | Völklingen | 1 |
| Helios Klinikum Berlin-Buch | Berlin | 1 |
| Evangelisches Krankenhaus Königin Elisabeth Herzberge gGmbH | Berlin | 1 |
| Vivantes Klinikum im Friedrichshain | Berlin | 1 |
| Bundeswehrkrankenhaus Berlin | Berlin | 1 |
| St. Hedwig-Krankenhaus Berlin | Berlin | 1 |
| Asklepios Klinikum Uckermark | Schwedt | 1 |
| Carl-Thiem-Klinikum Cottbus gGmbH | Cottbus | 1 |
| HELIOS Klinikum Bad Saarow | Bad Saarow | 1 |
| KMG Klinikum Güstrow | Güstrow | 1 |
| Sana HANSE-Klinikum Wismar GmbH | Wismar | 1 |
| Asklepios Klinik Pasewalk | Pasewalk | 1 |
| Dietrich-Bonhoeffer-Klinikum Standort Neubrandenburg | Neubrandenburg | 1 |
| Diakonissenkrankenhaus Dresden | Dresden | 1 |
| Paracelsus-Klinik Reichenbach | Reichenbach | 1 |
| ELBLANDKLINIKEN Stiftung & Co. KG, ELBLANDKLINIKUM Riesa | Riesa | 1 |
| Kreiskrankenhaus Freiberg | Freiberg | 1 |
| Universitätsklinikum Carl Gustav Carus Dresden an der Technischen Universität Dresden, Anstalt des öffentlichen Rechts des Freistaates Sachsen | Desden | 1 |
| Universitätsklinikum Leipzig Anstalt öffentlichen Rechts | Leipzig | 1 |
| Sana Kliniken Leipziger Land GmbH - Klinikum Borna | Borna | 1 |
| Johanniter-Krankenhaus Genthin-Stendal GmbH | Stendal | 1 |
| Universitätsklinikum Magdeburg A. ö. R. | Magdeburg | 1 |
| Carl-von-Basedow-Klinikum Saalekreis gGmbH | Merseburg | 1 |
| Evangelisches Krankenhaus Paul Gerhardt Stift | Luherstadt Wittenberg | 1 |
| HELIOS Klinik Lutherstadt Eisleben | Lutherstadt Eisleben | 1 |
| Dessau-Roßlau | Dessau-Roßlau | 1 |
| Kreiskrankenhaus Greiz GmbH | Greiz | 1 |
| Katholisches Krankenhaus "St. Johann Nepomuk" Erfurt | Erfurt | 1 |
| Südharz Klinikum Nordhausen gGmbH | Nordhausen | 1 |
| SRH Zentralklinikum Suhl GmbH | Suhl | 1 |
| Ilm-Kreis-Kliniken Arnstadt-Ilmenau gGmbH | Ilmenau | 1 |
