# Supplementary material for: Development of Incidence and Surgical Treatment of Penile Cancer in Germany from 2006 to 2016: Potential Implications for Future Management
Source: Ann Surg Oncol. 2021 Jun 12;28(13):9190–8. doi: 10.1245/s10434-021-10189-6 (PMC8591000; doi:10.1245/s10434-021-10189-6)
Supplement: Supplementary file 2 — Supplementary Table 2 Hospitals and caseload of inguinal LND for penile cancer for the year 2016. [file 10434_2021_10189_MOESM2_ESM.docx]

**Supplementary Table 1:** Hospitals and caseload of penile amputation for the year 2016.

| Name of hospital | City | Total caseload of penile amputation |
| --- | --- | --- |
| Chirurgische Klinik München-Bogenhausen | München | 55 |
| Universitätsklinikum Hamburg-Eppendorf | Hamburg | 35 |
| AGAPLESION MARKUS KRANKENHAUS | Frankfurt am Main | 34 |
| Universitätsmedizin Rostock - Teilkörperschaft der Universität Rostock | Rostock | 23 |
| Kliniken Essen-Mitte Evang. Huyssens-Stiftung 01 | Essen | 21 |
| Klinikum Stuttgart - Katharinenhospital (KH) und Olgahospital / Frauenklinik (OH) | Stuttgart | 17 |
| Asklepios Westklinikum Hamburg GmbH | Hamburg | 16 |
| Urologische Klinik München - Planegg | Planegg | 16 |
| Universitätsklinikum Freiburg | Freiburg | 15 |
| Klinikum Oldenburg AöR | Oldenburg | 12 |
| Paracelsus Klinik Düsseldorf Golzheim | Düsseldorf | 12 |
| Städtische Kliniken Neuss - Lukaskrankenhaus - GmbH | Neuss | 12 |
| Marien Hospital Herne, Klinikum der Ruhr-Universität Bochum | Herne | 12 |
| Universitätsklinikum Tübingen | Tübingen | 12 |
| Klinikum St. Georg gGmbH | Leipzig | 11 |
| Klinikum rechts der Isar der Technischen Universität München | München | 9 |
| UNIVERSITÄTSKLINIKUM Schleswig-Holstein, Campus Kiel | Kiel | 8 |
| Universitätsklinikum Münster | Münster | 8 |
| SLK-Kliniken Heilbronn GmbH - Klinikum am Gesundbrunnen | Heilbronn | 8 |
| Städtisches Klinikum Karlsruhe | Karlsruhe | 8 |
| Universitätsklinikum Mannheim GmbH | Mannheim | 8 |
| Klinikum Bamberg - Betriebsstätte am Bruderwald | Bamberg | 8 |
| St. Hedwig-Krankenhaus Berlin | Berlin | 8 |
| Zeisigwaldkliniken Bethanien Chemnitz | Chemnitz | 8 |
| Universitätsklinikum Köln | Köln | 7 |
| Klinikum Dortmund Nord | Dortmund | 7 |
| Universitätsmedizin der Johannes Gutenberg-Universität Mainz | Mainz | 7 |
| Universitätsklinikum Carl Gustav Carus Dresden an der Technischen Universität Dresden, Anstalt des öffentlichen Rechts des Freistaates Sachsen | Desden | 7 |
| Universitätsmedizin Göttingen | Göttingen | 6 |
| Augusta-Kranken-Anstalt gGmbH Bochum | Bochum | 6 |
| Diakonie Klinikum Ev. Jung-Stilling-Krankenhaus | Siegen | 6 |
| Krankenhaus Salem derEvang. Stadtmission Heidelberg gGmbH | Heidelberg | 6 |
| Schwarzwald-Baar Klinikum Villingen-Schwenningen GmbH | Villingen-Schwenningen | 6 |
| DONAUISAR Klinikum Deggendorf | Deggendorf | 6 |
| Caritas-Krankenhaus St. Josef | Regensburg | 6 |
| Klinikum Nürnberg Nord | Nürnberg | 6 |
| St. Bernward Krankenhaus | Hildesheim | 5 |
| Klinikum Bremen-Mitte | Bremen | 5 |
| Universitätsklinikum Düsseldorf | Düsseldorf | 5 |
| Evangelisches Klinikum Niederrhein gGmbH | Oberhausen | 5 |
| Universitätsklinikum Bonn | Bonn | 5 |
| Marien-Krankenhaus Bergisch Gladbach | Bergisch Gladbach | 5 |
| Franziskus Hospital Bielefeld | Bielefeld | 5 |
| Rems-Murr-Klinikum Winnenden | Winnenden | 5 |
| Klinikum der Universität München | München | 5 |
| Waldkrankenhaus St. Marien | Erlangen | 5 |
| Klinikum Augsburg mit Kliniken für Kinder und Jugendliche | Augsburg | 5 |
| SHG-Kliniken Völklingen | Völklingen | 5 |
| Universitätsklinikum des Saarlandes | Homburg | 5 |
| Charité - Universitätsmedizin Berlin | Berlin | 5 |
| Vivantes Klinikum Am Urban | Berlin | 5 |
| Universitätsmedizin Greifswald - Körperschaft des öffentlichen Rechts | Greifswald | 5 |
| Universitätsklinikum Halle (Saale) | Halle (Saale) | 5 |
| UNIVERSITÄTSKLINIKUM Schleswig-Holstein, Campus Lübeck | Lübeck | 4 |
| KRH Klinikum Siloah | Hannover | 4 |
| Borromäus Hospital Leer gGmbH | Leer | 4 |
| St. Elisabeth-Krankenhaus Köln GmbH | Köln | 4 |
| Herz-Jesu-Krankenhaus Hiltrup GmbH | Münster | 4 |
| Klinikum Westmünsterland St. Marien- Krankenhaus Ahaus | Ahaus | 4 |
| St. Josefs-Hospital | Dortmund | 4 |
| Gemeinschaftsklinikum Mittelrhein, Kemperhof | Koblenz | 4 |
| Klinikum Friedrichshafen GmbH | Friedrichshafen | 4 |
| Kreisklinik Ebersberg | Ebersberg | 4 |
| Klinikum Saarbrücken | Saarbrücken | 4 |
| Vivantes Klinikum im Friedrichshain | Berlin | 4 |
| Städtisches Klinikum Brandenburg GmbH | Brandenburg an der Havel | 4 |
| HELIOS Kliniken Schwerin | Schwerin | 4 |
| Evangelisches Krankenhaus Paul Gerhardt Stift | Luherstadt Wittenberg | 4 |
| Kath. Marienkrankenhaus gemeinnützige GmbH | Hamburg | 3 |
| GFO Kliniken Troisdorf, Betriebsstätte St. Josef Troisdorf | Troisdorf | 3 |
| Klinikum Fulda gAG | Fulda | 3 |
| Klinikum Ingolstadt GmbH | Ingolstadt | 3 |
| Krankenhaus Martha-Maria Nürnberg | Nürnberg | 3 |
| Universitätsklinikum Würzburg | Würzburg | 3 |
| Klinikum Ernst von Bergmann gemeinnützige GmbH | Potsdam | 3 |
| HELIOS Klinikum Erfurt | Erfurt | 3 |
| AK SEGEBERGER KLINIKEN GMBH | Bad Segeberg | 2 |
| Asklepios Klinikum Harburg | Hamburg | 2 |
| Städtisches Klinikum Braunschweig gGmbH | Braunschweig | 2 |
| Evangelisches Krankenhaus Göttingen-Weende gGmbH | Göttingen | 2 |
| Klinikum Wolfsburg | Wolfsburg | 2 |
| KRH Klinikum Robert Koch Gehrden | Gehrden | 2 |
| Helios Klinik Cuxhaven GmbH | Cuxhaven | 2 |
| AMEOS Klinikum Seepark Geestland | Geestland | 2 |
| Städtisches Klinikum Lüneburg gemeinnützige GmbH | Lüneburg | 2 |
| Bonifatius Hospital Lingen | Lingen | 2 |
| Klinikum Osnabrück GmbH | Osnabrück | 2 |
| Ammerland-Klinik Westerstede | Westerstede | 2 |
| DIAKO Ev. Diakonie-Krankenhaus gemeinnützige GmbH | Bremen | 2 |
| Universitätsklinikum Essen | Essen | 2 |
| Alfried Krupp Krankenhaus Steele | Essen | 2 |
| Evangelisches Krankenhaus Oberhausen GmbH | Oberhausen | 2 |
| [2017-09-27 17:15:22] Städtisches Klinikum Solingen gemeinnützige GmbH | Solingen | 2 |
| HELIOS Klinikum Wuppertal GmbH | Wuppertal | 2 |
| Johanniter GmbH - Waldkrankenhaus Bonn | Bonn | 2 |
| Kliniken der Stadt Köln gGmbH - Krankenhaus Holweide | Köln | 2 |
| St.-Antonius-Hospital | Eschweiler | 2 |
| Krankenhaus Düren gem. GmbH | Düren | 2 |
| Malteser Krankenhaus St. Hildegardis | Köln | 2 |
| St. Barbara-Hospital | Gladbeck | 2 |
| Prosper-Hospital gGmbH | Recklinghausen | 2 |
| St. Elisabeth-Hospital Beckum GmbH | Beckum | 2 |
| Knappschaftskrankenhaus Bottrop GmbH | Bottrop | 2 |
| Marien-Hospital Marl | Marl | 2 |
| Klinikum Herford | Herford | 2 |
| Brüderkrankenhaus St. Josef Paderborn | Paderborn | 2 |
| AGAPLESION ALLGEMEINES KRANKENHAUS HAGEN gem. GmbH | Hagen | 2 |
| St. Josef-Krankenhaus, Hamm Bockum-Hövel | Hamm | 2 |
| Asklepios Paulinen Klinik | Wiesbaden | 2 |
| Hochtaunus-Kliniken gGmbH - Bad Homburg | Bad Homburg | 2 |
| Kliniken des Main-Taunus-Kreises GmbH | Bad Soden | 2 |
| Universitätsklinikum Frankfurt | Frankfurt am Main | 2 |
| Klinikum Kassel GmbH | Kassel | 2 |
| [2017-07-13 14:21:21] Klinikum der Stadt Ludwigshafen am Rhein gGmbH | Ludwigshafen | 2 |
| Klinikum Ludwigsburg | Ludwigsburg | 2 |
| Kliniken Sindelfingen | Sindelfingen | 2 |
| Theresienkrankenhaus Mannheim | Mannheim | 2 |
| GRN Gesundheitszentren Rhein-Neckar gGmbH Kreiskrankenhaus Eberbach | Eberbach | 2 |
| Siloah St. Trudpert Klinikum | Pforzheim | 2 |
| Ortenau Klinikum Offenburg-Gengenbach Standort Ebertplatz | Offenburg | 2 |
| Barmherzige Brüder Krankenhaus München | München | 2 |
| Klinikum Landshut gGmbH | Landshut | 2 |
| Klinikum St. Elisabeth Straubing GmbH | Straubing | 2 |
| Klinikum Coburg GmbH | Coburg | 2 |
| Sana Klinikum Hof | Hof | 2 |
| Klinikum Fichtelgebirge gGmbH - Haus Marktredwitz | Marktredwitz | 2 |
| Klinikum Fürth | Fürth | 2 |
| Diakonie Klinikum Neunkirchen gemeinnützige GmbH | Neunkirchen | 2 |
| Vivantes Humboldt-Klinikum | Berlin | 2 |
| Ruppiner Kliniken | Neuruppin | 2 |
| Havelland Kliniken GmbH, Klinik Nauen | Nauen | 2 |
| Sana HANSE-Klinikum Wismar GmbH | Wismar | 2 |
| Dietrich-Bonhoeffer-Klinikum Standort Neubrandenburg | Neubrandenburg | 2 |
| Heinrich-Braun-Klinikum gemeinnützige GmbH, Standort Zwickau | Zwickau | 2 |
| Universitätsklinikum Leipzig Anstalt öffentlichen Rechts | Leipzig | 2 |
| Sana Kliniken Leipziger Land GmbH - Klinikum Borna | Borna | 2 |
| St. Elisabeth-Krankenhaus Leipzig | Leipzig | 2 |
| AMEOS Klinikum Aschersleben-Staßfurt GmbH | Aschersleben | 2 |
| Carl-von-Basedow-Klinikum Saalekreis gGmbH | Merseburg | 2 |
| KLINIKUM MAGDEBURG gemeinnützige GmbH | Magdeburg | 2 |
| Thüringen-Kliniken "Georgius Agricola" GmbH | Saalfeld | 2 |
| Südharz Klinikum Nordhausen gGmbH | Nordhausen | 2 |
| SRH Wald-Klinikum Gera GmbH | Gera | 2 |
| Diakonissenkrankenhaus Flensburg | Flensburg | 1 |
| Asklepios Nordseeklinik Westerland/Sylt | Sylt/OT Westerland | 1 |
| HELIOS Agnes Karll Krankenhaus Bad Schwartau | Bad Schwartau | 1 |
| Regio Kliniken GmbH - Klinikum Wedel | Wedel | 1 |
| Klinikum Itzehoe | Itzehoe | 1 |
| Sana Kliniken Lübeck GmbH | Lübeck | 1 |
| Asklepios Klinik St. Georg | Hamburg | 1 |
| Asklepios Klinik Barmbek | Hamburg | 1 |
| Asklepios Harzklinik Goslar | Goslar | 1 |
| HELIOS Albert-Schweitzer-Klinik Northeim | Northeim | 1 |
| HELIOS Klinikum Salzgitter GmbH | Salzgitter | 1 |
| Gesundheitseinrichtungen Hameln-Pyrmont GmbH - Sana Klinikum Hameln-Pyrmont | Hameln | 1 |
| DIAKOVERE Friederikenstift | Hannover | 1 |
| Medizinische Hochschule Hannover | Hannover | 1 |
| Vinzenzkrankenhaus Hannover | Hannover | 1 |
| Helios Kliniken Mittelweser | Nienburg | 1 |
| KRH Klinikum Großburgwedel | Burgwedel | 1 |
| Krankenhaus Buchholz | Buchholz in der Nordheide | 1 |
| ALLGEMEINES KRANKENHAUS CELLE | Celle | 1 |
| AGAPLESION DIAKONIEKLINIKUM ROTENBURG gemeinnützige GmbH | Rotenburg (Wümme) | 1 |
| Elbe Klinikum Stade | Stade | 1 |
| St. Franziskus-Hospital | Lohne | 1 |
| Helios Klinik Wesermarsch | Nordenham | 1 |
| Hümmling Hospital Sögel gGmbH | Sögel | 1 |
| Marien Hospital Düsseldorf GmbH | Düsseldorf | 1 |
| Helios St. Marien Klinik | Duisburg | 1 |
| HELIOS Klinikum Krefeld | Krefeld | 1 |
| Malteser Krankenhaus St. Josefshospital Uerdingen | Krefeld | 1 |
| [2017-10-02 11:07:06] Alexianer Krefeld GmbH - Krankenhaus Maria-Hilf | Krefeld | 1 |
| Städtische Kliniken Mönchengladbach GmbH | Mönchengladbach | 1 |
| Krankenhaus St. Franziskus | Mönchengladbach | 1 |
| St.-Clemens-Hospital Geldern | Geldern | 1 |
| Hospital zum Heiligen Geist Kempen GmbH & Co. KG | Kempen | 1 |
| Marien-Hospital Wesel gGmbH | Wesel | 1 |
| Katholisches Klinikum Essen - Marienhospital Altenessen | Essen | 1 |
| HELIOS Klinikum Niederberg | Velbert | 1 |
| Uniklinik RWTH Aachen | Aachen | 1 |
| Franziskushospital Aachen GmbH | Aachen | 1 |
| Malteser Krankenhaus Seliger Gerhard Bonn/Rhein-Sieg | Bonn | 1 |
| Klinikum Leverkusen gGmbH | Leverkusen | 1 |
| St.-Katharinen-Hospital GmbH | Frechen | 1 |
| Betriebsteil Bardenberg | Würselen-Bardenberg | 1 |
| St. Antonius-Hospital Gronau GmbH | Gronau | 1 |
| St. Vincenz-Krankenhaus | Datteln | 1 |
| Mathias-Spital Rheine / Gesundheitszentrum Rheine | Rheine | 1 |
| Bergmannsheil und Kinderklinik Buer GmbH | Gelsenkirchen | 1 |
| St. Elisabeth-Krankenhaus | Ibbenbüren | 1 |
| Evangelisches Krankenhaus Bielefeld gGmbH - Standort Johannesstift | Bielefeld | 1 |
| Klinikum Gütersloh | Gütersloh | 1 |
| St. Ansgar Krankenhaus Höxter | Höxter | 1 |
| Klinikum Lippe Detmold | Detmold | 1 |
| Krankenhaus Lübbecke-Rahden, Betriebsstelle Lübbecke | Lübbecke | 1 |
| Johannes Wesling Klinikum Minden | Minden | 1 |
| HELIOS Klinikum Schwelm | Schwelm | 1 |
| Ev. Krankenhaus Witten gGmbH | Witten | 1 |
| Städt. Krankenhaus Maria-Hilf Brilon gGmbH | Brilon | 1 |
| Klinikum Lüdenscheid, Märkische Kliniken GmbH | Lüdenscheid | 1 |
| St. Josefs-Hospital Lennestadt | Lennestadt | 1 |
| Kreisklinikum Siegen GmbH | Siegen | 1 |
| Marien-Hospital | Erwitte | 1 |
| Knappschaftskrankenhaus Dortmund, Klinikum Westfalen GmbH | Dortmund | 1 |
| Katholische Kliniken im Märkischen Kreis - St. Elisabeth Hospital | Iserlohn | 1 |
| Klinikum Darmstadt GmbH | Darmstadt | 1 |
| Klinikum Frankfurt Höchst GmbH | Frankfurt am Main | 1 |
| Krankenhaus Nordwest | Frankfurt am Main | 1 |
| Sankt Katharinen Krankenhaus GmbH | Frankfurt | 1 |
| Klinik Maingau vom Roten Kreuz | Frankurt | 1 |
| Universitätsklinikum Gießen und Marburg, Standort Gießen | Gießen | 1 |
| Klinikum Wetzlar-Braunfels | Wetzlar | 1 |
| Sana Klinikum Offenbach GmbH | Offenbach | 1 |
| HELIOS Dr. Horst-Schmidt-Kliniken Wiesbaden | Wiesbaden | 1 |
| Dill Kliniken | Dillenburg | 1 |
| Krankenhaus Gelnhausen | Gelnhausen | 1 |
| Krankenhaus Eichhof Lauterbach | Lauterbach | 1 |
| Universitätsklinikum Gießen und Marburg GmbH, Standort Marburg | Marburg | 1 |
| Stadtkrankenhaus Korbach gGmbH | Korbach | 1 |
| GPR Klinikum | Rüsselsheim | 1 |
| AGAPLESION Evangelisches Krankenhaus Mittelhessen | Gießen | 1 |
| Asklepios Stadtklinik Bad Wildungen | Bad Wildungen | 1 |
| Kassel | Kassel | 1 |
| Krankenhaus Maria Hilf | Bad Neuenahr-Ahrweiler | 1 |
| Gemeinschaftsklinikum Mittelrhein, St. Elisabeth Mayen | Mayen | 1 |
| Evangelisches Krankenhaus, Betriebsstätte Dierdorf | Dierdorf | 1 |
| Marienhaus Klinikum St. Elisabeth Neuwied | Neuwied | 1 |
| Cusanus Krankenhaus Bernkastel-Kues | Bernkastel-Kues | 1 |
| Krankenhaus der Barmherzigen Brüder Trier | Trier | 1 |
| Westpfalz-Klinikum GmbH - Standort I Kaiserslautern | Kaiserslautern | 1 |
| Krankenhaus Hetzelstift | Neustadt an der Weinstra&szlig;e | 1 |
| Städtisches Krankenhaus Pirmasens gGmbH | Pirmasens | 1 |
| Klinikum Worms gGmbH | Worms | 1 |
| Klinikum Landau-Südliche Weinstraße - Klinik Bad Bergzabern | Bad Bergzabern | 1 |
| Diakonie-Klinikum Stuttgart | Stuttgart | 1 |
| medius KLINIK OSTFILDERN-RUIT | Ostfildern | 1 |
| Karl-Olga-Krankenhaus GmbH | Stuttgart | 1 |
| Klinikum Mittelbaden Baden-Baden Balg | Baden-Baden | 1 |
| Fürst-Stirum-Klinik Bruchsal | Bruchsal | 1 |
| Diakonissenkrankenhaus Mannheim | Mannheim | 1 |
| Kliniken Nagold | Nagold | 1 |
| Loretto-Krankenhaus (RkK) Freiburg | Freiburg | 1 |
| Kreiskrankenhaus Emmendingen | Emmendingen | 1 |
| Hegau-Bodensee-Klinikum Singen | Singen | 1 |
| St. Elisabethen-Krankenhaus gGmbH | Lörrach | 1 |
| Klinikum am Steinenberg | Reutlingen | 1 |
| Universitätsklinikum Ulm | Ulm | 1 |
| St. Elisabethen-Klinikum | Ravensburg | 1 |
| RoMed Klinikum Rosenheim | Rosenheim | 1 |
| Asklepios Stadtklinik Bad Toelz | Bad Tölz | 1 |
| Kreiskliniken des Landkreises Mühldorf a. Inn GmbH - Klinik Mühldorf | Mühldorf am Inn | 1 |
| Klinikum Starnberg | Starnberg | 1 |
| Klinikum Traunstein | Traunstein | 1 |
| Städtisches Klinikum München GmbH, Klinikum Harlaching | München | 1 |
| Städtisches Klinikum München GmbH, Klinikum Bogenhausen | München | 1 |
| Isar Kliniken GmbH | München | 1 |
| Rotkreuzklinikum München | München | 1 |
| Klinikum Passau | Passau | 1 |
| Krankenhaus Grafenau | Grafenau | 1 |
| Kliniken Nordoberpfalz AG - Klinikum Weiden | Weiden i. d. OPf. | 1 |
| Klinikum Neumarkt | Neumarkt i.d.OPf. | 1 |
| Sana Kliniken des Landkreises Cham - Krankenhaus Cham | Cham | 1 |
| Klinikum Kulmbach | Kulmbach | 1 |
| ANregiomed Klinikum Ansbach | Ansbach | 1 |
| Leopoldina-Krankenhaus | Schweinfurt | 1 |
| Juliusspital Würzburg | Würzburg | 1 |
| Main-Klinik Ochsenfurt gGmbH | Ochsenfurt | 1 |
| Klinikum Aschaffenburg-Alzenau | Aschaffenburg | 1 |
| die stadtklinik im diako | Augsburg | 1 |
| Memmingen | Memmingen | 1 |
| [2017-09-26 13:29:38] Rotkreuzklinik Lindenberg gemeinnützige GmbH | Lindenberg | 1 |
| Knappschaftsklinikum Saar GmbH, Krankenhaus Sulzbach | Sulzbach | 1 |
| CaritasKlinikum Saarbrücken Standort St. Theresia | Saarbrücken | 1 |
| Helios Klinikum Berlin-Buch | Berlin | 1 |
| Evangelisches Krankenhaus Königin Elisabeth Herzberge gGmbH | Berlin | 1 |
| BG-Unfallklinik - Unfallkrankenhaus Berlin gGmbH | Berlin | 1 |
| Bundeswehrkrankenhaus Berlin | Berlin | 1 |
| Asklepios Klinikum Uckermark | Schwedt | 1 |
| Klinikum Barnim GmbH, Werner Forßmann Krankenhaus | Eberswalde | 1 |
| DRK Krankenhaus Luckenwalde | Luckenwalde | 1 |
| Evangelisches Krankenhaus Luckau gGmbH | Luckau | 1 |
| Carl-Thiem-Klinikum Cottbus gGmbH | Cottbus | 1 |
| Klinikum Niederlausitz GmbH | Lauchhammer | 1 |
| Lausitz Klinik Forst GmbH | Forst | 1 |
| Klinikum Frankfurt (Oder) GmbH | Frankfurt (Oder) | 1 |
| HELIOS Klinikum Bad Saarow | Bad Saarow | 1 |
| KMG Klinikum Güstrow | Güstrow | 1 |
| Asklepios Klinik Pasewalk | Pasewalk | 1 |
| Diakonissenkrankenhaus Dresden | Dresden | 1 |
| Paracelsus-Klinik Reichenbach | Reichenbach | 1 |
| ELBLANDKLINIKEN Stiftung & Co. KG, ELBLANDKLINIKUM Riesa | Riesa | 1 |
| Städtisches Klinikum Dresden - Standort Friedrichstadt | Dresden | 1 |
| Kreiskrankenhaus Freiberg | Freiberg | 1 |
| HELIOS Klinikum Aue | Aue | 1 |
| HELIOS Vogtland-Klinikum Plauen | Plauen | 1 |
| Johanniter-Krankenhaus Genthin-Stendal GmbH | Stendal | 1 |
| HELIOS Bördeklinik | Oschersleben / OT Neindorf | 1 |
| Universitätsklinikum Magdeburg A. ö. R. | Magdeburg | 1 |
| Krankenhaus Martha-Maria Halle-Dölau | Halle (Saale) | 1 |
| HELIOS Klinik Lutherstadt Eisleben | Lutherstadt Eisleben | 1 |
| Dessau-Roßlau | Dessau-Roßlau | 1 |
| Asklepios Klinik Weißenfels | Weißenfels | 1 |
| Krankenhaus St. Marienstift Magdeburg GmbH | Magdeburg | 1 |
| AMEOS Klinikum Halberstadt | Halberstadt | 1 |
| Kreiskrankenhaus Greiz GmbH | Greiz | 1 |
| Katholisches Krankenhaus "St. Johann Nepomuk" Erfurt | Erfurt | 1 |
| HELIOS Klinik Blankenhain | Blankenhain | 1 |
| HELIOS Klinikum Gotha | Gotha | 1 |
| SRH Zentralklinikum Suhl GmbH | Suhl | 1 |
| Eichsfeld Klinikum gGmbH | Kleinbartloff OT Reifenstein | 1 |
| Ilm-Kreis-Kliniken Arnstadt-Ilmenau gGmbH | Ilmenau | 1 |
